# Analysis of Psilocybin-Assisted Therapy in Medicine: A Narrative Review

**DOI:** 10.7759/cureus.21944

**Published:** 2022-02-05

**Authors:** Shawn Ziff, Benjamin Stern, Gregory Lewis, Maliha Majeed, Vasavi Rakesh Gorantla

**Affiliations:** 1 Department of Anatomical Sciences, St. George’s University School of Medicine, St. George’s, GRD

**Keywords:** psychoactive drug, selective serotonin reuptake inhibitors, mood disorders, depression, addiction, psilocybin, psychedelics

## Abstract

Psilocybin-containing mushrooms have been consumed by various cultures in many different parts of the world for thousands of years. Psilocybin, a classic psychedelic, contains unique psychoactive properties and has been incorporated into religious ceremonies and investigated for its medicinal value. In the mid-20th century, psilocybin, along with most other classic psychedelics (5HT-2A agonists), was classified as a Schedule I substance, bringing a halt to research on its medicinal utility. The resurgence of clinical trials involving psilocybin in the 21st century has produced promising results concerning the treatment of addiction, depression, and end-of-life mood disorders. Results from these trials have shown significant reductions in depression and anxiety when compared with a placebo, and one trial found no significant difference when compared to a routinely prescribed selective serotonin reuptake inhibitor (SSRI). Studies conducted with patients with advanced-stage cancer have demonstrated that psilocybin may also be beneficial at reducing depression and anxiety associated with psychological crises due to a terminal diagnosis. Psilocybin therapy in the treatment of addiction, which is notoriously difficult to treat, has shown encouraging results. Due to its low toxicity and low risk of overuse, psilocybin has the potential to have a significant influence in the field of addiction medicine. Psilocybin addiction research has been primarily focused on nicotine and alcohol and, in a few small, open-label trials, has shown superiority over traditional therapies. Psilocybin has a relatively unique and incompletely understood mechanism of action, which allows it to be given at several isolated periods. This infrequent dosing regimen has been shown to produce durable effects with minimal toxicity. This review analyzes the potential of psilocybin in the treatment of addiction, depression, and end-of-life mood disorders. In addition, it will discuss the difficulties involved with conducting scientific research on psychedelic compounds, adverse effects, and the therapeutic measures that are necessary to accompany the safe and effective administration of these psychoactive chemicals.

## Introduction and background

The use of psychedelic compounds in the treatment of illness may appear unconventional at first glance; however, this application is not a new phenomenon. Many cultures have been using psychoactive plants for thousands of years to treat and diagnose medical ailments [1]. Evidence of the use of mescaline, a serotonin 5-hydroxytryptamine 2A (5HT-2A) receptor agonist similar to psilocybin, has been recovered by archeologists in Texas and radiocarbon dated to 5700 years ago [2]. The ability of psychoactive plants to produce both spiritual and medicinal effects has placed them in a unique category among many societies. Throughout their history, they have been considered both holy and immoral, revered and criminalized. The legality of and public opinion on these compounds have fluctuated; however, the compounds themselves have largely remained the same. Many compounds that fit this description are placed into a category called the 5HT-2A agonists, otherwise known as “classic hallucinogens.” While many of these chemicals share similar effects and have the same potential for medicinal value, we will focus this paper on one, namely, psilocybin, the active chemical found in psilocybe “magic” mushrooms.

Psilocybin-containing mushrooms can grow all over the world and appear to be ubiquitous across many cultures [1]. Evidence of the use of neurotropic fungi exists from Northwest Mexico, dating over 2000 years old. However, cave paintings from Spain depicting a bull and what appears to be neurotropic mushrooms (which commonly grow on cow and bull manure) have been dated back to between 6000 and 8000 years ago [3]. The inclusion of mushrooms in ancient cultural artifacts shows the importance that these societies placed on these fungi.

In the early 20th century, psilocybin and lysergic acid diethylamide (LSD) became an intriguing new topic to study among medical professionals in psychiatry. Albert Hofman, who famously synthesized lysergic acid diethylamide (LSD) while working for Sandoz in 1938 and then again in 1943, was also responsible for first isolating and synthesizing psilocybin in 1958, the psychoactive chemical found in psilocybe mushrooms [1]. These compounds, which are often categorized as the “classic hallucinogens,” were used with increasing frequency until their classification as Schedule I substances under the UN Convention on Drugs in 1967 [1]. Additionally, following the UN’s classification, many countries included their own laws for restricting or regulating the use of these substances.

Since their inclusion in the Schedule I class of substances, there has been an ongoing discussion about the benefit and harm that these compounds produce and how they should be considered within the greater society. Recently, there has been a resurgence of evidence-based research and clinical trials involving these compounds. “This is a wonderful, fruitful time for discovery because people are suddenly willing to consider these substances as therapeutics again, which hasn’t happened in 50 years,” said Jennifer Mitchell, a neuroscientist at the University of California, San Francisco. Dr. Mitchell is the lead author of a study exploring the benefits of treating patients suffering from severe post-traumatic stress disorder (PTSD) with 3,4-methylenedioxymethamphetamine (MDMA), a psychoactive chemical.

While it is certain that more research is necessary to determine how best to utilize psilocybe mushrooms and the active ingredient psilocybin, many potential uses have been proposed and experiments conducted. Among those include the treatment of addiction, depression, and end-of-life mood disorders. Throughout this paper, we will review and discuss studies conducted that test psilocybin’s potential ability to treat these ailments. By assessing these areas of medicine and contrasting the current treatment options with psilocybin-assisted therapy, we aim to determine how efficacious these new methods are. In addition, we will consider the secondary effects and adverse effects of all treatment options for a more holistic review.

## Review

Mechanism of action

A group of compounds that have recently sparked interest for their treatment potential is psychedelic drugs including psilocybin, dimethyltryptamine (DMT), mescaline, and lysergic acid diethylamide (LSD), among others [4]. These drugs are currently controversial for their hallucinogenic effects and recreational use; however, their resemblance to tryptophan enables them to act as serotonin agonists and activate 5HT receptors, mainly the 5HT-2A receptor [5]. This effect makes them promising candidates in the treatment of substance abuse disorders as well as mood disorders [6]. This is due to the fact that substance use disorder (SUD) is the manifestation of several neurophysiological mechanisms including the dopaminergic pathways of the striatum (reward center), 5-HT pathways connecting with the striatum, and the corticotropin-releasing hormone (CRH) pathway, which drives the hypothalamic-pituitary-adrenal (HPA) axis [7].

One of the hallucinogens of interest in the treatment of mental health disorders is tryptamine: psilocybin. This compound can be isolated from over a hundred species of mushroom and is thus a naturally occurring alkaloid [8]. Most of the psilocybin-containing species of mushrooms come from the *Psilocybe* genus [8]. In its natural form, psilocybin or 3-[2-(dimethylamino) ethyl]-1H-indol-4-yl dihydrogen phosphate is a prodrug that is converted in the body to psilocin or 4-hydroxy-N,N-dimethyltryptamine [9]. The structure of psilocybin is such that it cannot cross the blood-brain barrier. Its metabolite psilocin, however, is more lipophilic and therefore more active [6]. The active metabolite can be found 20-40 minutes after ingestion as psilocybin is quickly converted to its metabolite in the stomach acid or via first-pass metabolism with a bioavailability of around 50% and a half-life of 2.5 hours [6].

This metabolite is the active form of psilocybin and closely resembles the structure of serotonin [10]. The psychedelic is, therefore, able to activate 5HT receptors as mentioned, ultimately leading to their downregulation [4]. In such instances, there is a compensatory upregulation in the metabotropic glutamate receptors (mGluR2/3) due to the inverse relationship between mGluR2/3 and 5HT-2A receptors [5]. The stimulation of 5HT-2A receptors on pyramidal cells in levels V and VI in the cortex by psilocin, a metabolite, leads to the release of glutamate in the prefrontal cortex [6].

The significance of the upregulation of the mGluR2/3 pathway is the result of the receptor’s abundance in the route from the medial prefrontal cortex to the nucleus accumbens where these receptors play a role in the mediation of craving and addiction, as well as cognitive function [5]. It has been found that persons with an alcohol addiction have a deficiency or downregulation in their mGluR2 pathway in the infralimbic region of the medial prefrontal cortex [5].

On the other hand, psilocybin’s effects at the 5HT receptors are responsible for decreased depression and suicidal behaviors, as well as increased memory and learning [11]. Further, it is believed that psilocin’s activation of the 5HT-2A receptor is responsible for increased release of dopamine from the striatum, which is capable of regulating the defective reward pathway in patients who suffer from depression and with suicidal ideation [6].

**Figure 1:**
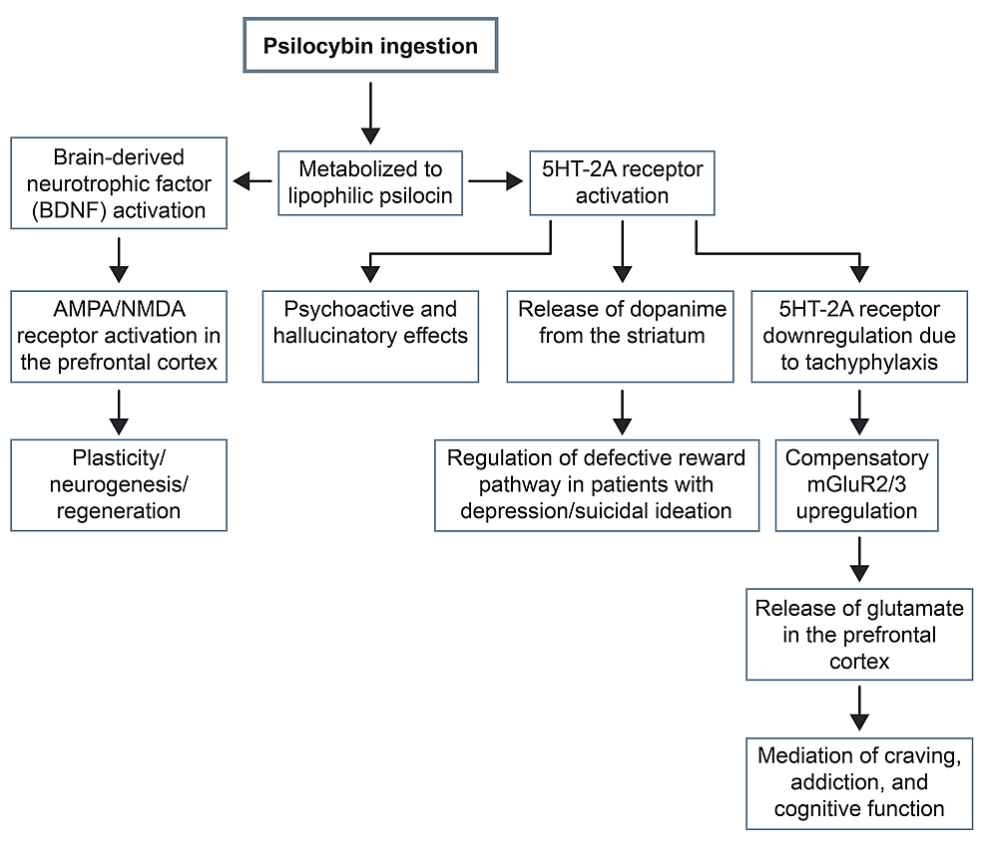
Psilocybin mechanism of action. mGluR2/3: metabotropic glutamate receptors; AMPA: α-amino-3-hydroxy-5-methyl-4-isoxazole propionic acid; NMDA: N-methyl-D-aspartate; BDNF: brain-derived neurotrophic factor; 5HT-2A: 5-hydroxytryptamine 2A

In addition to its effects on the serotonin receptors, psilocybin is able to activate G protein-coupled receptors via brain-derived neurotrophic factor (BDNF) and enable downstream effects such as signal transduction and gene expression [9]. These changes are potentially responsible for plasticity, regeneration, and neurogenesis. Psilocybin-mediated BDNF is also responsible for the activation of N-methyl-D-aspartate (NMDA) and α-amino-3-hydroxy-5-methyl-4-isoxazole propionic acid (AMPA) receptors in the prefrontal cortex, leading to increased plasticity (Figure 1) [9].

Use in Addiction Treatment

Among the proposed therapeutic applications of psilocybin is its use in the treatment of substance use disorders. The current FDA-approved pharmacotherapies for substance use disorder (SUD) have modest effect sizes, and patients often relapse [12]. Psychedelic therapy models for addiction treatment were heavily researched between 1950 and 1970. However, research in this field came to a halt with the restriction of the classic psychedelics (LSD, psilocybin, DMT, etc.) as Schedule I controlled substances in 1970 [13]. A renewed enthusiasm for this area emerged in the past decade with early clinical trials of psilocybin showing promising results in treating nicotine dependence and alcohol dependence. Additional phase II trials are currently underway.

A number of characteristics distinguish psilocybin as a useful pharmacotherapy in the treatment of substance use disorders. First, psilocybin has a low abuse potential despite its classification as a Schedule I controlled substance. Tolerance to the reinforcing effects builds rapidly in users who take psilocybin repeatedly [13]. However, signs of physical dependence on psilocybin and withdrawal have not been documented [7]. Second, the half-life of psilocin, the active metabolite, is three hours [14]. The peak effect typically occurs within 60-90 minutes of oral ingestion, which subsides over the following six hours after ingestion [15]. This pharmacokinetic profile makes psilocybin more attractive as a pharmacologic treatment than other psychedelics with slower elimination and longer durations of action [13]. Third, psilocybin is associated with few adverse effects, most of which are mild, including mild, transient hypertension and tachycardia, mild headaches, and rarely prolonged psychosis [16]. Fourth, the duration of therapeutic effect in the context of SUD is such that only two or three administrations of psilocybin may have long-term or even permanent beneficial effects [17].

Nicotine Addiction

The enduring personal meaning and spiritual awakening effects of psilocybin have been put to use in the context of smoking cessation. A small, open-label study conducted through Johns Hopkins University incorporated two to three psilocybin administrations to examine its utility in smoking cessation [16]. This study enrolled 15 participants for the 15-week course of treatment. The participants received cognitive behavioral therapy (CBT) for smoking cessation as well as preparation for psilocybin administration weekly for the first four weeks. The participants’ target quit date (TQD) coincided with their treatment session for week 5 in which they received their first dose of psilocybin of 20 mg/70 kg. The subjects continued to meet with study staff weekly and received a second psilocybin dose in the seventh week and a third optional dose in the 13th week. The second and third doses were higher at 30 mg/70 kg, but the participants could repeat the initial 20 mg/70 kg dose instead if they preferred [16]. The biological markers of smoking abstinence were employed throughout the study and at long-term follow-up in the form of breath CO and urine cotinine [16]. Each marker served as biological verification of abstinence. The participants also completed a Mysticism Scale questionnaire at intake, one week after the second psilocybin session, and one week after the third psilocybin session when applicable [16].

The study found that 12 of 15 participants (80%) were abstinent at the six-month follow-up [16]. Three of these 12 had suffered lapses between the end of the 15-week course and the six-month follow-up, but all three resumed smoking abstinence prior to the follow-up meeting as verified by biological markers. The participants also indicated that the psilocybin experiences changed their “orientation toward the future, so that long-term benefits outweighed immediate desires (73%)” and that psilocybin “strengthened participants’ belief in their ability to quit (73%)” [16].

The results of this open-label study were remarkable when contrasted with those of the current FDA-approved smoking cessation pharmacotherapies. One such therapy, sustained-release bupropion, yields seven-day point prevalence abstinence rates of 24.9% at six months post-target quit date, while varenicline shows 33.5% abstinence rates at six months post-target quit date [18], both of which are less than half the rates seen in this psilocybin study. Another method of smoking cessation treatment that consisted of a combination of bupropion, nicotine replacement, and cognitive behavioral therapy (CBT) for 12 weeks duration yielded a 40% abstinence rate at six months [19]. This multipronged approach, while more effective than pharmacotherapy alone, does not boast the 80% six-month abstinence rate seen with this small psilocybin study.

A long-term follow-up study was performed on the original participants of the study. At the 12-month follow-up, 10 of the 12 participants who returned were smoking abstinent as confirmed by biological marker tests. Of these 10, eight reported continuous abstinence since their target quit date (TQD) (60%) [17]. The most effective smoking cessation medications demonstrate about 30% abstinence at 12 months post-TQD [19]. At long-term follow-up (mean: 30 months post-TQD), nine participants were biologically confirmed abstinent with seven of them reporting having not smoked since TQD [17]. The results of this study led to a larger clinical trial examining the efficacy of single-dose psilocybin versus an eight- to 10-week course of nicotine replacement therapy with both groups receiving the same CBT smoking cessation treatment [17]. This trial is currently underway.

Alcohol Use Disorder

A small, open-label study with 10 participants was conducted to examine the utility of psilocybin in the treatment of alcohol use disorder [20]. All 10 individuals had a diagnosis of alcohol dependence according to the Diagnostic and Statistical Manual of Mental Disorders, Fourth Edition (DSM-IV) criteria. The participants underwent a 12-week course of treatment consisting of seven sessions of motivational enhancement therapy (MET). The first psilocybin administration consisted of a 0.3 mg/kg dose. The participants received a dose of 0.4 mg/kg for the second psilocybin administration with the exception of one participant who repeated the same initial dose [20].

The percent heavy drinking days (defined as five or more drinks in one day for males and four or more in one day for females) of the participants decreased a mean of 26% compared to their baselines in the remainder of the study following the first psilocybin session [20]. Additionally, the percent of days when subjects consumed any alcohol decreased a mean of 27% compared to their baseline following the first psilocybin session. Another notable result from this study was a statistically significant decrease in Penn Alcohol Craving Scale (PACS) values from weeks 8-12 compared to baseline values [20]. The PACS is a widely used assessment tool to measure craving for alcohol. Its validity and reliability have been well demonstrated [21]. The adverse effects of psilocybin treatment consisted of five participants reporting mild headaches lasting less than one day and one participant reporting nausea with one episode of vomiting. None of the participants required pharmacologic intervention for adverse effects [20]. Although this study was small and lacked a control group, the findings are encouraging, and an adequately powered, double-blind trial is currently underway [22].

Applications in Other Substance Use Disorders

The potential benefits of psilocybin in the area of substance use disorders and addiction are currently being examined in several clinical trials. The encouraging results of small, open-label studies revealed that there is a promise with nicotine and alcohol addiction. This concept has been extrapolated to methamphetamine use disorder with a small clinical trial comparing therapy and two psilocybin sessions versus the standard rehabilitation treatment with a total of about 30 participants [23]. Another current clinical trial is comparing the benefits of one psilocybin session versus diphenhydramine as a control for the treatment of cocaine use [24]. This trial is unique in that it is placebo-controlled, an invaluable component of pharmaceutical research that is difficult when dealing with psychedelic substances. Another application of psilocybin in a small clinical trial is examining the effect of psilocybin with guided counseling in addition to buprenorphine/naloxone maintenance therapy in patients with opioid use disorder [25]. The results of these small studies (Table 1), in addition to the larger phase II studies regarding nicotine and alcohol addiction previously mentioned, may have large implications for the field of substance abuse therapy.

**Table 1:** Use of psilocybin in the treatment of addiction to nicotine, alcohol, methamphetamine, cocaine, and opioids. CBT: cognitive behavioral therapy; AUD: alcohol use disorder: MET: motivational enhancement therapy; OUD: opioid use disorder

Substance	Study design	Participants	Treatment regimen	Results	Follow-up
Nicotine [16]	Open-label, pilot study	15 adult smokers	CBT, 20 mg/70 kg psilocybin at fifth week, 30 mg/70 kg psilocybin at seventh week, optional 30 mg/70 kg psilocybin at 13^th^ week	80% of participants were abstinent at six months, 60% of participants were continuously abstinent through six months	60% of participants were continuously abstinent at 12 months, 75% of participants were abstinent at long-term follow-up (mean: 30 months) [17]
Alcohol [20]	Open-label, pilot study	10 adults with AUD	MET, 0.3 mg/kg psilocybin, 0.4 mg/kg (or 0.3 mg/kg if preferred by the participant) psilocybin	Compared to baseline, 26% mean decrease in percent heavy drinking days following first psilocybin session, 27% mean decrease in the percent of days when subjects consumed alcohol, a significant decrease in PACS value	None to date
Methamphetamine [23]	Interventional, randomized trial	30 adults with amphetamine-related disorders	Experimental group: six-week psychotherapy protocol, 25 mg, and 30 mg psilocybin administrations two weeks apart; control group: treatment-as-usual while admitted to an inpatient rehabilitation program	Pending; estimated completion date: June 30, 2024	Pending
Cocaine [24]	A double-blinded, randomized, controlled trial	40 adults with cocaine-related disorders	Experimental group: 0.36 mg/kg psilocybin; comparator group: 100 mg diphenhydramine	Pending; estimated completion date: April 2022	Pending
Opioids [25]	Open-label, pilot study	10 adults with OUD	Two administrations of psilocybin with guided counseling, approximately four weeks apart	Pending; estimated completion date: August 2022	Pending

Use in Major Depression

Major depressive disorder is a growing problem within our society, with roughly 20% of people experiencing some form of depression within their lifetime [26]. The current first-line treatment for major depressive disorder is a treatment with a class of medications called SSRIs [27]. However, these drugs can take weeks to begin working, have adverse side effects, and often have limited efficacy, with 30%-50% of patients being unresponsive to treatment and 10%-30% considered resistant to treatment entirely [28]. Due to the inconsistent efficacy in some individuals of current treatment options, alternative methods are constantly being investigated.

Psilocybin, an agonist acting on the serotonin 5-hydroxytryptamine 2A (5HT-2A) receptor, has been implicated as a potential treatment option for a variety of illnesses, depression being one of them [27]. In this approach, the patient consumes psilocybin in a therapeutic setting, guided by a trained professional referred to as a therapist. Psilocybin-assisted therapy is a unique form of treatment because it does not require daily administration. Rather, it consists of two or three doses given several weeks apart. Research is currently underway to determine the long-term efficacy of this approach to treatment.

An experiment conducted at the Center for Psychedelic and Consciousness Research at Johns Hopkins Bayview Medical Center tested the efficacy of psilocybin in treating major depressive disorder. The team used a randomized, waiting list controlled trial to assess their patients’ results. Twenty-four patients were included in the study; 13 patients were in the immediate treatment group, receiving 20 mg/70 kg of psilocybin during session one and 30 mg/70 kg during session two, at week 3 and 4, respectively, and 11 patients were in the delayed group, receiving the same dosage as the immediate groups, at weeks 11 and 12, respectively. The delayed group allowed for the comparison between two otherwise similar groups, one having undergone the psilocybin therapy sessions and one not having completed it yet [28].

Using the GRID-Hamilton Depression Rating Scale (GRID-HAMD), researchers were able to compare the effects of psilocybin on the immediate treatment group compared with that of the delayed group, with the higher score indicating more severe depression. They found that while the delayed treatment group’s scores did not change significantly within the first 11 weeks, the immediate treatment group’s scores dropped significantly following the psilocybin therapy, measured in week 5. The mean GRID-HAMD scores one week after psilocybin treatment and four weeks after treatment were significantly lower for the immediate group (8.0 at week 1 and 8.5 at week 4) than that of the delayed group (23.8 at week 5 and 23.5 at week 8, which had yet to undergo treatment.

The difference in depression scores between the two groups can be attributed to the psilocybin therapy and is further supported by the secondary measures using the Quick Inventory of Depressive Symptomatology-Self-Report (QIDS-SR). Using the QIDS-SR depression rating scale, they found that the levels of depression fell from 16.7 (which was the baseline for the immediate group) to 6.3 one week after the treatment with psilocybin (Cohen d = 2.6; 95% confidence interval (CI): 1.8-3.5; P < 0.001) [28]. The conclusion of the study found that 71% of the participants had a significant reduction in their depression, measured as a greater than 50% reduction in the GRID-HAMD score at week 1 and 4 following treatment, and 58% and 54% of the participants were considered in remission (GRID-HAMD < 7) after week 1 and 13, respectively [28].

In a study conducted at the British National Institute for Health Research (NIHR) Imperial Clinical Research Facility, psilocybin was tested against escitalopram, an SSRI used for the treatment of moderate to severe major depressive disorder. Using a double-blind, randomized, controlled trial, the team found that there was no statistically significant difference in the antidepressant effects of psilocybin versus escitalopram after administering both for six weeks, two 25 mg doses of psilocybin three weeks apart, and daily escitalopram [27]. This trial used the QIDS-SR-16 as the primary method to assess a participant’s level of depression. The scale ranged from 0 to 27, with higher numbers indicating higher levels of depression. Both treatment options lead to a decrease in the QIDS-SR-16 score. The psilocybin group had a reduction of 8 ± 1 points, and the escitalopram group had a reduction of 6 ± 1 points. The difference was 2 points, with a 95% CI of -5.0 to 0.9 and a P value of 0.17 [27].

The trial included several secondary measures of depression such as the Beck Depression Inventory 1A (BDI-1A), 17-Item Hamilton Depression Rating Scale (HAM-D-17), and the Montgomery and Asberg Depression Rating Scale (MADRS). These measures tended to support the use of psilocybin over escitalopram; however, the confidence intervals were not corrected for these comparisons [27]. While they were unable to suggest that one treatment was superior to the other, they established that they both decreased the depression scores in their respective cohorts. The conclusion that there was no significant difference between the two treatments reflects the potential for psilocybin as a treatment option. Additional research is required to correct for secondary factors, and future studies will be better able to provide more inclusive comparisons.

Use in Cancer/End-of-Life Mood Disorders

It is common for patients with a terminal diagnosis such as cancer to develop mood disorders such as depression and anxiety [29]. The current treatment options for these patients are limited, complicated by the long list of medications the patient is on, and often do not produce adequate results. Psilocybin was initially investigated as a potential treatment for mood disorders in terminally ill patients from the 1950s to the 1970s; however, due to its addition to the list of scheduled substances, most research halted in the 1970s [30]. In recent years, psilocybin research has been renewed and shown as a potential therapeutic for end-of-life depression and anxiety.

In a recent double-blind, placebo-controlled study, psilocybin was tested in 12 patients with various forms of cancer, to reduce the psychological burden of a terminal diagnosis [30]. Each patient had advanced-stage cancer and a clinical diagnosis of at least one of the following: acute stress disorder, generalized anxiety disorder, anxiety disorder due to cancer, or adjustment disorder with anxiety. In this study, each patient underwent both the placebo and active treatment administered several weeks apart. The order of which was randomized. The placebo consisted of 250 mg of niacin, due to its ability to cause mild flushing, but no psychological effects, and the active treatment consisted of a relatively small dose of 0.2 mg/kg psilocybin [30]. The results were calculated using various types of self-questionnaires. The Beck Depression Inventory (BDI), Profile of Mood States (POMS), and State-Trait Anxiety Inventory (STAI) were measured the day before each session, the day after each session, two weeks after each session, and once per month for six months following the final session. In addition, the POMS, STAI, Five-Dimension Altered States of Consciousness profile (5D-ASC), and Brief Psychiatric Rating Scale were given directly following the session. The intervals at which these tests were given helped identify the cause of any potential changes in scores.

The mean BDI score of the psilocybin group dropped 6.1 points after the treatment, from 16.1 one day before treatment to 10.0 at the two-week follow-up. A reduction in the BDI by roughly 30% was shown at the one-month follow-up after the psilocybin treatment, and this trend continued and was shown to be significant at the six-month follow-up (P = 0.03) [30]. This represents a significant decrease in self-reported depression among the participants. The placebo group did not show any significant reduction between one day before and at the two-week follow-up.

The POMS showed a similar decrease in mean scores between one day before psilocybin and the two-week follow-up, demonstrating significant improvement in mood after a psilocybin treatment. No differences in mean scores were observed one day before the placebo and the two-week follow-up. A paired post hoc test showed that the mean scores one day before the psilocybin treatment were significantly higher than that of the placebo, regardless of the order of the two treatments. This aberration may account for some of the differences; however, the results were sustained for the six-month follow-up period, so it is unlikely to be entirely due to this anomaly [30]. The STAI did not show any significant decrease in anxiety in the two weeks following either treatment. However, at the one-month follow-up, there was a sustained decrease in anxiety scores, which persisted through the six-month follow-up.

A separate study, consisting of 51 individuals, used a double-blind crossover design with two sessions to compare the efficacy of a small dose of psilocybin versus a larger dose in treating patients with cancer with clinical depression and anxiety [29]. The participants received either a large dose of psilocybin (22 or 30 mg/70 kg) or a low dose (1 or 3 mg/70 kg). The low dose was expected to have negligible psychoactive effects. The study utilized a selection of self-questionnaires and community observer questionnaires completed by family, friends, or colleagues.

The outcomes of this study showed that the larger dose of psilocybin contributed to a greater reduction of depression and anxiety during the five-week period prior to the crossover. At the six-month interval, the STAI, Hamilton Depression Rating Scale, Hamilton Anxiety Rating Scale, and BDI all demonstrated a sustained decrease in the score, indicating less severe symptoms. Of the participants, 80% had a clinically significant reduction in their depression and anxiety at the six-month follow-up, and 60% of the participants were in remission, meaning they no longer qualified as having a diagnosis [31].

It is not fully understood how psilocybin achieves these results; however, it is proposed that the consciousness-altering properties are partly responsible due to the correlation between larger doses and more substantial outcomes. However, the authors found that a dose greater than 30 mg/70 kg more often led to psychologically challenging experiences and were thus not beneficial [29]. The exact mechanism by which psilocybin achieves these favorable outcomes requires additional study.

Discussion

Analysis of recent and ongoing studies show that psilocybin has promising therapeutic effects. Psilocybin has been implemented as a potential therapy for hard-to-treat disorders such as addiction, depression, and end-of-life anxiety. There are still questions about how best to proceed with psilocybin-assisted therapy, and further studies are necessary. However, if it was determined to effectively and safely treat these diseases, it would benefit millions of people and greatly reduce suffering throughout the world.

Relationship With Therapy

Clinical trials involving psilocybin have shown promising results in reducing the burden of many psychiatric conditions. However, psilocybin and other psychoactive chemicals currently being studied require more than simply administering a capsule and waiting for the beneficial effect. This often includes weeks of therapy leading up to the first dose of psilocybin and again directly following. In a recent paper describing the goals and challenges of psychedelic therapists, Phelps identifies three core phases of treatment: “preparation for the medicine-assisted session, the medicine session itself, and integration of the psychological material that arises during preparation and the medicine session” [32].

Another important aspect to control for is set and setting. Set refers to the patient’s and therapist’s goals for the session, often discussed in detail during therapy beforehand. It includes the motivating factors, intentions, and expectations. In addition, it includes the specific type of guidance the therapist will use throughout the experience. The setting is used to describe the physical, mental, and emotional environment of the patient before and during the treatment [32]. There are differing opinions on how best to prepare an individual for a psychedelic-assisted therapy session; however, most recent studies use similar environments. These often include a quiet room with the patient lying down, a choice of preset music to listen to, and a blindfold, which is often encouraged.

As more studies are being conducted and clinical trials begin, developing these ancillary procedures will become an essential part of the overall treatment. There will be a growing demand for trained therapists able to administer psilocybin therapy. It will be prudent to continue to research and understand the supporting measures that lead to the most effective outcomes.

Relationship Between Mechanism of Action and Efficacy

Much has been learned about psilocybin in the past few decades; however, the mechanism of action responsible for the therapeutic results is still not completely understood. Promising new studies are using MRI technologies in order to understand the effects of psilocybin on resting neurological pathways [31]. During an interview in 2019, Dr. Griffiths, who is a lead researcher of psilocybin at Johns Hopkins, stated, “Neuroimaging studies have shown that at the time psilocybin is given, there’s a lot of neural connectivity among areas of the brain that usually don’t talk to one another.” He also said, “And this occurs for the duration of time that the psilocybin is in the system. It has led to a hypothesis that this may open a window of neuroplasticity in which there may be a rewiring going on.” This is a striking difference in mechanism of action to the majority of therapeutics currently in use, suggesting that the beneficial effects are a result of “rewiring of the brain” and that the altered consciousness experience of the individual leads to lasting durable changes after minimal treatments.

Various studies demonstrate that a greater level of mystical experiences was associated with and predicted better outcomes [28]. This strengthens the assertion that psilocybin works in part by changing the way we think and view the world. The ability of psilocybin to alter an individual’s perception may be linked to changes in mood and behavior that last longer than the duration of the drug itself and thus lead to longer-lasting changes. This could provide patients with an alternative to daily medications and allow them to seek treatment at much longer intervals.

Observable Changes in Personality After Psychedelic Administration

Psilocybin has been shown to change one’s personality. In a small study, participants with moderate to severe treatment-resistant depression were administered a 10 mg and 25 mg dose of psilocybin one week apart. Using the Revised NEO Personality Inventory (NEO-PI-R), a baseline personality score was assessed pretreatment and then assessed again three months posttreatment. When assessing the five personality traits, it was found that there was a significant increase in openness and extraversion scores with a significant decrease in neuroticism scores. Conscientiousness scores showed trend-level increases with no change to agreeableness scores. The positive personality changes were correlated with the patient’s degree of insightfulness during the psilocybin-induced experience [33].

The positive changes seen in personality with psychedelics are not limited to patients with preexisting mental conditions. In one study, 20 healthy participants were assessed at baseline with a Revised Life Orientation Test (LOT-R) and a NEO-PI-R personality test. Two weeks post-administration of 75 μg LSD, patients reported a significant increase in optimism with LOT-R and openness with NEO-PI-R. The study also shows a trend toward an increase in the personality trait agreeableness [34].

These studies, along with others, illuminate a growing body of evidence that psychedelic compounds cause positive personality modification. It was found that healthy participants benefit from the use of these compounds, as well as participants with preexisting cognitive conditions. This finding further broadens the discussion about the potential use of psychedelic compounds, both for treating diagnosed conditions and in healthy individuals.

Adverse Effects

As research opens to explore the therapeutic options for these compounds, it is equally important to explore the potential downsides. Psilocybin is considered a Schedule I controlled substance, categorized as a drug that has high abuse potential with no clinical application. When administering psilocybin or any psychoactive compound, there is a potential to elicit psychologically challenging situations, sometimes referred to as a “bad trip.” These can include delirium, panic attacks, depersonalization, extreme distress, and other symptoms similar to schizophrenia [35]. In controlled settings, some participants experienced elevated blood pressure and gastrointestinal distress including nausea and vomiting [29]. It is important to note that these side effects are transient. One of the more serious concerns when using psilocybin is eliciting the onset of schizophrenia. In the majority of test subjects, there is little evidence to support that symptoms of psychosis persist after psilocybin use. When psychotic illness does occur, it is thought to be due to the expression of a predisposition rather than the drug creating the disorder [36]. Due to this, patient screening is important to reduce the occurrence of adverse drug effects.

Limitations With Testing Psychedelic Compounds

Performing strict scientific research on psychedelics comes with many challenges. First comes the problem of performing an effective double-blind study. In a study using a self-controlled blinding system, it was discovered that most patients and facilitators were able to assess which treatment options were administered due to the consciousness-altering nature of psilocybin [30]. Some studies use placebos such as niacin, which have mild flushing effects, or intravenous saline [34]. However, even with these placebos, more research and testing are required to find a suitable and effective form of blinding.

Expectations of what will happen during the drug-induced experience may affect the efficacy of the compounds being studied. When gaining informed consent, the participants are made aware that they may experience out-of-body sensations, depersonalization, or severe anxiety. This, in combination with prior expectations and environmental factors, may make the participant more sensitive to the context of the experience [34]. Biases are likely to occur in some of these studies due to the relatively small sample size as well as selection bias when using participants willing to take a Schedule I drug [37].

Set and setting also act as an important factor in the experience of the participant. Early experiments on psychedelics were performed in sterile laboratory rooms with minimal interaction with the patient in an attempt to control for variables. These experiments often had poor outcomes due to the elicitation of psychologically challenging situations brought on in part by the environment. Recent experiments have built upon these past models and modified the environment to provide a more suitable setting. The creation of an ideal set and setting is subject to ongoing research and may change from patient to patient and also as researchers continue to learn more about the best practices for administration.

Research on the classic psychedelics originally focused on treating end-of-life cancer-related distress [31]; however, recent studies have expanded the therapeutic scope of psilocybin to include major depressive disorder and addiction, among others. Recent studies have demonstrated that psilocybin is efficacious at lowering the reported levels of depression in individuals with and without a terminal diagnosis. This would prove beneficial to millions of people who suffer from depression and are unresponsive or resistant to treatment. Self-reported levels of depression show significant reductions following psilocybin treatments when compared to that of placebos. In addition, when compared to escitalopram, a commonly prescribed SSRI for major depression, psilocybin showed no statistical difference in efficacy and improvements in some nonsignificant secondary measures.

The use of psilocybin in the treatment of addiction could alter the landscape of an area of medicine that has evolved incrementally [7]. A national survey performed in 2017 found that nearly 50 million Americans aged 12 or older were current cigarette smokers, with nearly 28 million of those smoking daily [38]. While the percentage of smokers in the United States has declined in the past several decades, tobacco use still accounts for a large portion of morbidity, as well as an economic burden in the United States [39]. Similarly, alcohol use is associated with a large degree of morbidity and economic burden as well [40]. The same national survey from 2017 found that 16.7 million Americans were heavy drinkers, defined as binge drinking on five or more days in the past month [38]. For those individuals with substance use disorders and tobacco addiction who wish to decrease or eliminate their behavior, psilocybin-assisted therapy may become a useful asset for medical professionals attending to these individuals. Furthermore, the use of a substance with a low risk of dependence and toxicity on a limited, supervised basis in the pharmacologic treatment of individuals predisposed to addiction is ideal. The results of current clinical trials are likely to enhance the understanding of the efficacy of psilocybin-assisted addiction treatment (Table 2).

**Table 2:** Benefits of psilocybin treatment in addiction, major depressive disorder, and end-of-life mood disorders compared with challenges with experimentation of these compounds. GRID-HAMD: GRID-Hamilton Depression Rating Scale; QIDS-SR-16: Quick Inventory of Depressive Symptomatology–Self-Report; BDI: Beck Depression Inventory 1A

Benefits of treatment	Challenges with experimentation
Nicotine and alcohol addiction – Results: 80% of the patients had smoking cessation at six months [16], 26% decrease in heavy drinking, and 27% decrease in days participants consumed alcohol [20]	Adverse reactions: delirium, panic attacks, depersonalization, extreme distress, elevated blood pressure, nausea, vomiting, and other symptoms similar to schizophrenia [29,35]
Major depressive disorder – Results: 71% and 54% of patients had a reduction of depression at one week and 13 weeks posttreatment, respectively, using GRID-HAMD [28], and the treatment group had a reduction of 8 ± 1 points compared with the escitalopram group with a reduction of 6 ± 1 points using QIDS-SR-16 [27]	Double-blinding: even with the administration of activated placebos (e.g., niacin), patients are often aware of which treatment they receive [30]
End-of-life mood disorder – Results: Using BDI, the psilocybin group had a 6.1 drop in self-reported depression compared with the control after one-week and six-month follow-up [30], and 80% of the patients experienced a clinically significant drop in depression and anxiety at six months posttreatment [31]	Small sample size: biases are likely to occur in some of these studies due to the relatively small sample size, as well as selection bias when using participants willing to take a Schedule I drug [37]

## Conclusions

Psilocybin offers a wide range of possible medical applications, according to clinical studies. Addiction medicine, depression, and end-of-life mood disorders are among the areas with the most evidence of benefit. The traditional treatments for these disorders are frequently ineffective, and psilocybin-assisted therapy might provide a new treatment option for millions of patients. The results of the first clinical trials are encouraging, albeit there have been some setbacks. For future studies, it will be critical to improve testing procedures. When it comes to mind-altering medications such as psilocybin, conducting thorough double-blind experiments is challenging, and subject selection biases are a concern. Despite these difficulties, psilocybin continues to show promise in the treatment of certain mental illnesses. In many of the trials listed above, it showed statistically significant benefits while also having fewer negative side effects than standard drugs. Psilocybin is still regarded as harmful by many people because of its complicated cultural and legal past, and its acceptance as a treatment will take some time. As fresh research on psilocybin emerges, it becomes obvious that psilocybin-assisted therapy has demonstrated potential as a therapeutic aid in the treatment of some psychiatric diseases.

## References

[1] Rucker JJ, Iliff J, Nutt DJ (2018). Psychiatry & the psychedelic drugs. Past, present & future. Neuropharmacology.

[2] Bruhn JG, De Smet PA, El-Seedi HR, Beck O (2002). Mescaline use for 5700 years. Lancet.

[3] Akers BP, Francisco Ruiz J, Piper A, Ruck CA (2011). A prehistoric mural in Spain depicting neurotropic Psilocybe mushrooms. Econ Bot.

[4] Mahapatra A, Gupta R (2017). Role of psilocybin in the treatment of depression. Ther Adv Psychopharmacol.

[5] Meinhardt MW, Pfarr S, Fouquet G (2021). Psilocybin targets a common molecular mechanism for cognitive impairment and increased craving in alcoholism. Sci Adv.

[6] Strumila R, Nobile B, Korsakova L (2021). Psilocybin, a naturally occurring indoleamine compound, could be useful to prevent suicidal behaviors. Pharmaceuticals (Basel).

[7] de Veen BT, Schellekens AF, Verheij MM, Homberg JR (2017). Psilocybin for treating substance use disorders?. Expert Rev Neurother.

[8] Gotvaldová K, Hájková K, Borovička J, Jurok R, Cihlářová P, Kuchař M (2021). Stability of psilocybin and its four analogs in the biomass of the psychotropic mushroom Psilocybe cubensis. Drug Test Anal.

[9] Heuschkel K, Kuypers KP (2020). Depression, mindfulness, and psilocybin: possible complementary effects of mindfulness meditation and psilocybin in the treatment of depression. A review. Front Psychiatry.

[10] Patra S (2016). Return of the psychedelics: psilocybin for treatment resistant depression. Asian J Psychiatr.

[11] Zeiss R, Gahr M, Graf H (2021). Rediscovering psilocybin as an antidepressive treatment strategy. Pharmaceuticals (Basel).

[12] Bogenschutz MP, Johnson MW (2016). Classic hallucinogens in the treatment of addictions. Prog Neuropsychopharmacol Biol Psychiatry.

[13] Johnson MW, Griffiths RR, Hendricks PS, Henningfield JE (2018). The abuse potential of medical psilocybin according to the 8 factors of the Controlled Substances Act. Neuropharmacology.

[14] Brown RT, Nicholas CR, Cozzi NV (2017). Pharmacokinetics of escalating doses of oral psilocybin in healthy adults. Clin Pharmacokinet.

[15] Lowe H, Toyang N, Steele B (2021). The therapeutic potential of psilocybin. Molecules.

[16] Johnson MW, Garcia-Romeu A, Cosimano MP, Griffiths RR (2014). Pilot study of the 5-HT2AR agonist psilocybin in the treatment of tobacco addiction. J Psychopharmacol.

[17] Johnson MW, Garcia-Romeu A, Griffiths RR (2017). Long-term follow-up of psilocybin-facilitated smoking cessation. Am J Drug Alcohol Abuse.

[18] Gonzales D, Rennard SI, Nides M (2006). Varenicline, an alpha4beta2 nicotinic acetylcholine receptor partial agonist, vs sustained-release bupropion and placebo for smoking cessation: a randomized controlled trial. JAMA.

[19] Laude JR, Bailey SR, Crew E (2017). Extended treatment for cigarette smoking cessation: a randomized control trial. Addiction.

[20] Bogenschutz MP, Forcehimes AA, Pommy JA, Wilcox CE, Barbosa PC, Strassman RJ (2015). Psilocybin-assisted treatment for alcohol dependence: a proof-of-concept study. J Psychopharmacol.

[21] Hartwell EE, Bujarski S, Green R, Ray LA (2019). Convergence between the Penn Alcohol Craving Scale and diagnostic interview for the assessment of alcohol craving. Addict Behav Rep.

[22] National Library of Medicine. (2014, June June (2022). ClinicalTrials.gov: A double-blind trial of psilocybin-assisted treatment of alcohol dependence. https://clinicaltrials.gov/ct2/show/NCT02061293.

[23] National Library of Medicine. (2022, January January (2022). ClinicalTrials.gov: Psilocybin-enhanced psychotherapy for methamphetamine use disorder. https://clinicaltrials.gov/ct2/show/NCT04982796.

[24] National Library of Medicine. (2015, May May (2022). ClinicalTrials.gov: Psilocybin-facilitated treatment for cocaine use. https://clinicaltrials.gov/ct2/show/NCT02037126.

[25] National Library of Medicine. (2021, January January (2022). ClinicalTrials.gov: Adjunctive effects of psilocybin and buprenorphine. https://clinicaltrials.gov/ct2/show/NCT04161066.

[26] Malhi GS, Mann JJ (2018). Depression. Lancet.

[27] Carhart-Harris R, Giribaldi B, Watts R (2021). Trial of psilocybin versus escitalopram for depression. N Engl J Med.

[28] Davis AK, Barrett FS, May DG (2021). Effects of psilocybin-assisted therapy on major depressive disorder: a randomized clinical trial. JAMA Psychiatry.

[29] Griffiths RR, Johnson MW, Carducci MA (2016). Psilocybin produces substantial and sustained decreases in depression and anxiety in patients with life-threatening cancer: a randomized double-blind trial. J Psychopharmacol.

[30] Grob CS, Danforth AL, Chopra GS, Hagerty M, McKay CR, Halberstadt AL, Greer GR (2011). Pilot study of psilocybin treatment for anxiety in patients with advanced-stage cancer. Arch Gen Psychiatry.

[31] Johnson MW, Griffiths RR (2017). Potential therapeutic effects of psilocybin. Neurotherapeutics.

[32] Phelps J (2017). Developing guidelines and competencies for the training of psychedelic therapists. J Humanist Psychol.

[33] Erritzoe D, Roseman L, Nour MM, MacLean K, Kaelen M, Nutt DJ, Carhart-Harris RL (2018). Effects of psilocybin therapy on personality structure. Acta Psychiatr Scand.

[34] Carhart-Harris RL, Kaelen M, Bolstridge M (2016). The paradoxical psychological effects of lysergic acid diethylamide (LSD). Psychol Med.

[35] Passie T, Halpern JH, Stichtenoth DO, Emrich HM, Hintzen A (2008). The pharmacology of lysergic acid diethylamide: a review. CNS Neurosci Ther.

[36] Reiff CM, Richman EE, Nemeroff CB (2020). Psychedelics and psychedelic-assisted psychotherapy. Am J Psychiatry.

[37] Goldberg SB, Pace BT, Nicholas CR, Raison CL, Hutson PR (2020). The experimental effects of psilocybin on symptoms of anxiety and depression: a meta-analysis. Psychiatry Res.

[38] Substance Abuse and Mental Health Services Administration (2018). Key substance use and mental health indicators in the United States: Results from the 2017 National Survey on Drug Use and Health (HHS Publication No. SMA 18-5068, NSDUH Series H-53). https://www.samhsa.gov/data/sites/default/files/cbhsq-reports/NSDUHFFR2017/NSDUHFFR2017.pdf.

[39] Centers for Disease Control and Prevention. (2020, April 28 (2022). Centers for Disease Control and Prevention: Health effects. https://www.cdc.gov/tobacco/basic_information/health_effects/index.htm.

[40] Centers for Disease Control and Prevention. (2021, January 14 (2022). Centers for Disease Control and Prevention: Deaths from excessive alcohol use in the United States. https://www.cdc.gov/alcohol/features/excessive-alcohol-deaths.html.

